# “*There Was a Light Bulb Moment*”: Using Persona Dyads to Empathise, Explore and Develop Person‐Centred Solutions in Mental Health Service Co‐Design

**DOI:** 10.1111/hex.70797

**Published:** 2026-08-04

**Authors:** A. Dominello, S. Lu, A. De Los Santos, M. Murphy, R. Clay‐Williams, C. Cheek

**Affiliations:** ^1^ Australian Institute of Health Innovation Sydney New South Wales Australia; ^2^ NSW Health Sydney New South Wales Australia; ^3^ University of Technology Sydney Sydney New South Wales Australia

**Keywords:** emergency departments, empathy, equity, experience‐based co‐design (EBCD), participatory method, patient and public involvement, priority populations

## Abstract

**Introduction:**

Co‐design of health services is an increasingly used method to meaningfully enable patient and public involvement, especially when working with priority populations whose perspectives are often overlooked in traditional top‐down approaches. However, differences in power and influence among stakeholders can lead to asymmetry and the marginalisation of certain voices. We used persona dyads (patient and provider) to co‐design improvements in Emergency Department care for people presenting with mental health concerns.

**Methods:**

Persona dyads were used in two Experience‐Based Co‐Design workshops with patients and providers. Transcripts were analysed to understand how personas were used, when and by whom. Participants completed surveys to evaluate the persona dyads and their co‐design experience.

**Results:**

Persona dyads humanised and contextualised Emergency Department experiences, fostering trust and safety by shifting attention from participants' personal histories to fictional representations without requiring personal disclosure. In the workshops, participants used the personas to establish shared understanding, enhance communication and generate creative, person‐centred solutions responsive to both sides of the care relationship. We observed differences in persona use across the workshops; there was greater reliance on the personas as participants navigated more complex patient and professional dynamics. The persona dyads facilitated empathy through not only presenting the patient but also the provider, a perspective often under‐represented in co‐design.

**Conclusion:**

Personas can be a useful person‐centred, participatory tool in health service co‐design, with dyads being particularly useful for cultivating empathy, levelling power, amplifying lived experience and creating safe opportunities for priority populations to contribute meaningfully to service improvement.

**Patient or Public Contribution:**

Individuals with lived experience of mental health concerns and Emergency Department healthcare providers were actively involved in the study. People with lived experience contributed to the co‐design of the persona dyads and participated in the workshop sessions. One author, who has lived experience of mental illness, provided critical input into the design and facilitation of the workshops, with particular emphasis on creating psychologically safe spaces for participation. Two authors are Emergency Department healthcare providers and study co‐investigators. The patient and provider contributors were involved in validating the findings, and ensuring interpretations were grounded in lived and professional experience.

## 1 Introduction

Patient and public involvement (PPI) in healthcare research is widely regarded as essential, though perspectives vary on what involvement should entail and why it should happen. Greenhalgh et al., [1] outlined three key arguments for PPI. First, patients and families have a right to contribute to and shape research that affects their lives. Second, involving patients and the public can enhance the quality, efficiency and real‐world relevance of research. Third, meaningful alliances with patients and the public strengthen the accountability and transparency of research processes and can improve the ability of projects to attract resources. PPI authenticates patients as epistemic experts and privileges experiences as stories to be shared [2, 3]. Involvement of people who experience social disadvantage and inequalities, referred to in this paper as ‘priority populations’, is fundamental for improving health, wellbeing and addressing perverse health disparities. We use the term ‘priority populations’, drawn from Australian and international health equity policy, to refer to groups who experience systematic and avoidable health inequities and whose perspectives are frequently under‐represented in service design, including, for example, people with mental health conditions, First Nations peoples, culturally and linguistically diverse communities and people with low socioeconomic status [4, 5]. Co‐design of health services is an increasingly used method to meaningfully enable PPI in an inclusive participatory space [6].

Co‐design is a collaborative design methodology that engages diverse stakeholders throughout the design process, drawing on their expertise and lived experiences to generate solutions that are contextually relevant and person‐centred [7]. Co‐design is especially valuable when working with priority populations, whose perspectives are often overlooked in traditional top‐down approaches [8]. Differences in power and influence among stakeholders, however, can lead to asymmetry and the marginalisation of certain voices [9]. This challenge is particularly pronounced in mental health, where histories of trauma, stigma and uneven power distribution are common [10]. Although co‐design has emerged as a leading framework for mental health service reform, creating a safe space for patients, carers and providers to understand problems and develop solutions can be difficult [10, 11].

Experience‐Based Co‐Design (EBCD) is one approach that has been applied across diverse healthcare settings, including a community forensic facility [12], acute hospitals [13], cancer services [14, 15], emergency departments [16, 17] and intensive care [14]. The aim of EBCD is to understand the subjective emotions, knowledge and experiences of service users, carers and providers to inform service improvements [18, 19]. In healthcare settings, EBCD commences with observations of the clinical environment and interviews with service users (patients) and providers to identify moments of shared concern, called touchpoints [20]. Patient interviews, traditionally filmed and edited to illustrate key moments in patient experiences [21], are used at the beginning of co‐design sessions to engender empathy in providers that ‘triggers’ momentum for change. By centring the voices of people with lived experience, these films are designed to have a powerful humanising effect among service providers [21]. As a participatory tool, trigger films support reflection, emotional connection and shared priority setting [20, 22].

In mental health contexts in particular, there are ethical and safety concerns regarding the collection and use of filmed patient interviews including re‐traumatisation, consent, confidentiality and stigma [18, 22, 23, 24]. Filmed interviews also pose a risk to participant anonymity if the film is preserved for reuse [20]. There is flexibility in how EBCD is applied, with research teams adapting the methodology to suit specific contexts and generate deep understanding [22]. In our study, which employed EBCD to improve Emergency Department (ED) services for people presenting with mental health concerns [25], we developed personas (fictional characters informed by real experiences) as an alternative to traditional patient trigger films. To preserve the function of trigger films to humanise experience, while also attending to participant safety, we created persona dyads, paired patient and provider personas, to represent the perspectives of both care recipients and care providers.

The use of persona dyads in co‐design consisted of three parts. In Part 1, we constructed two clinical scenarios from touchpoints revealed in analysis of data from non‐participant observations and interviews with mental health providers, patients and epistemic experts [26, 27]. Scenario 1 focussed on creating ways to preserve interpersonal care for patients with mental health needs in the ED who often experience a long wait to be seen by the mental health team. Scenario 2 focused on ideating a compassionate integrated model of care in the ED for adults presenting with, or at risk of, severe behavioural disturbance. In Part 2, we designed fictional patient/provider persona dyads from analysis of semi‐structured interviews with six healthcare providers and four patients with mental health concerns. Three overarching themes from the interviews: *Humanise*, acknowledging the human behind the patient or provider; *Contextualise*, situating the patient and provider in the healthcare system; and *Illuminate*, revealing thoughts, feelings and goals of the personas, guided the co‐designed persona dyad cards [28] (Box 1). This paper reports the findings from Part 3, application of these persona dyads in two co‐design workshops.

Box 1 **Persona dyads.**

**Table:** 

Scenario 1.		
Ana (fictional patient) presented to an ED feeling overwhelmed and scared, and faced a long wait in a crowded ED waiting area. Darius was the ED nurse (fictional provider) monitoring Ana, and had observed Ana's distress, knew that there was a chance she would leave the ED before being seen, and was considering how best to help her within his busy role.		
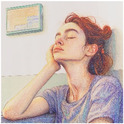 Ana.		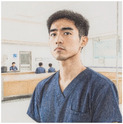 Darius.
Scenario 2.		
Bill (fictional patient) presented to an ED acutely intoxicated and agitated which he attributed to using ‘grass and ice’. Gina was the ED doctor (fictional provider) leading Bill's care, who noticed Bill's agitation but was also concerned about appropriately safeguarding the care team as well as other patients in proximity.		
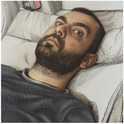 Bill.		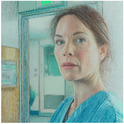 Gina.

## 2 Methods

### 2.1 Study Setting

This study is a component of a larger EBCD project that aimed to improve care for medically underserved communities presenting to three Emergency Departments (EDs) in a health district in NSW, Australia [25], with the study settings described in full elsewhere [26].

### 2.2 Participants

A purposive strategy for co‐design workshop recruitment was adopted. Patient participants were recruited if they had attended a study ED for mental health concerns and carers were recruited if they had supported a family member who attended an ED for mental illness. Healthcare professionals were eligible to participate if they worked in a study ED and cared for adults presenting with a mental illness. Patient participants could select to attend in‐person group or individual online co‐design sessions. All participants had to be able to provide valid consent and converse in English. Written consent was obtained from all participants.

### 2.3 Workshops 1 and 2

Workshop 1 commenced with a facilitator introducing participants to the clinical scenario and the Ana/Darius persona dyad. This was followed by:

- Activity 1, participant exploration of the needs of Ana (individual activity with feedback to the large group);
- Activity 2, a creative/group building exercise conducted in small groups;
- Activity 3, ideation and definition of ED service solutions (small group activity with feedback to the large group); and
- Activity 4, prioritisation and prototype of solutions (large group activity).

The Workshop 2 program, which used the Bill/Gina persona dyad, included:

- Activity 1, a creative exercise in small groups that was designed to extend participants' thinking beyond what was currently possible, followed by facilitator introduction to the clinical scenario and Bill/Gina persona dyad;
- Activity 2, participant exploration of the needs of Bill and Gina (small group activity with feedback to the large group); and
- Activity 3, ideation and prototype of ED service solutions (large group activity).

We used an activity before each small group discussion as a way to get the patient and provider participants talking with each other, and to move participants from their default patient or provider frame of reference towards more creative thinking. Each activity was deliberately designed so that it could not be achieved in a literal sense and therefore required more creative, lateral thinking. In Workshop 1 this was Activity 2 and in Workshop 2 it was Activity 1, in each case delivered before the small group discussion.

Each workshop was led by a facilitator. In Workshop 1, facilitation was led by C.C., an academic with experience in co‐design methods and a clinical background in nursing. Workshop 2 was scripted by C.C. but facilitated by R.C.‐W. due to illness. R.C.‐W. is an academic with experience in co‐design and human factors research. The workshops were designed in consultation with patients and providers to promote psychologically safe facilitation. Other research team members (three in Workshop 1 and four in Workshop 2) attended the workshops to assist with participant greeting, consent collection, small group discussion activities, and support in the event any individual became upset and needed to leave the workshop. In workshop analyses, we refer to all research team members as ‘facilitators’.

### 2.4 Data Collection

There were five forms of data collected and used in this analysis:

1. Participant demographic details.
2. Workshop audio recordings.
3. Two surveys completed anonymously by workshop participants at the conclusion of each workshop and online individual session:
  a. a bespoke evaluation survey about the persona dyads (refer Supporting Information S1: Appendix 1), comprising 15 closed‐ended questions: a 3‐pt nominal scale (Yes/No/Uncertain) was used for six items; a 5‐pt Likert scale (ranging from 1 (Very unhelpful) to 5 (Very helpful)) for four items; and a 4‐pt nominal scale (Too much, Just right, Too little, Uncertain) for two items, and 13 free text questions.
  b. the Public and Patient Engagement Evaluation Tool (PPEET) participant questionnaire for one‐time engagements [29], comprising 15 closed‐ended questions using a 5‐pt Likert scale (ranging from 1 (Strongly disagree) to 5 (Strongly agree)), and six free text questions.

4. Personal observation notes documented by workshop facilitators following each workshop on non‐verbal communication and group interaction.
5. Patient and provider co‐authors' reflections on how the persona dyads supported their involvement in the co‐design workshops.

### 2.5 Analysis of Workshop Data

Participant demographics were aggregated but because of small participant numbers are described in narrative form to preserve anonymity. Workshop audio‐recordings were transcribed verbatim, anonymised and analysed in Microsoft Excel. Each discussion point was ascribed a chronological timecode and analysed as a unique data point. Content analysis of group workshop transcripts was completed to determine persona use (by whom and when). Deductive analysis of manifest transcript content was undertaken to understand how personas were used by workshop participants. This analysis advanced through four sequential steps:

i. *a priori* codes were derived from the findings of a scoping review that explored the use of personas in health service co‐design [30].
ii. Codes were piloted with a sample of the transcripts and refined by three authors (A.D., R.C.‐W., C.C.) (refer Supporting Information S2: Appendix 2).
iii. Transcripts were double‐coded by two authors (A.D. and C.C.).
iv. Divergent coding was resolved through discussion.

Participant quotes provided in the results are not individually attributed to maintain the anonymity of individuals, however the participant quotes used represent three patients and six of the provider participants. Survey results were aggregated and analysed in Microsoft Excel using descriptive statistics. Facilitator observations were anonymised and reviewed to highlight non‐verbal moments that indicated participant engagement or workshop atmosphere. Patient and provider co‑author reflections were incorporated to provide reflexive insight into the use of persona dyads from those directly involved in co‑design.

We employed the Standards for Reporting Qualitative Research (SRQR) Guideline [31] (Supporting Information S3: Appendix 3), and documented patient involvement activities using GRIPP2‐SF (Guidance for Reporting Involvement of Patients and the Public ‐ Short Form) [32] (Supporting Information S4: Appendix 4). As a study that both describes a co‐design method and offers an early evaluation of it, our reporting draws on the SRQR and GRIPP2‐SF in a complementary way, as neither standard fully captures this hybrid focus.

### 2.6 Reflexivity Statement

The authors bring a wide range of professional and personal perspectives, including academia, nursing and lived experiences. The first author (A.D.) is a PhD candidate examining consumer involvement in co‑design. The research team's expertise covers human factors and resilience, emergency medicine, mental health, clinical practice, health services research, consumer involvement, co‑design, and implementation science, drawing on both qualitative and quantitative methodologies. Reflexivity was maintained throughout the project, with the first author engaging in regular reflective discussions on research processes and emerging findings with supervisors (R.C.‑W. and C.C.).

## 3 Results

Participants in the two co‐design workshops were patients (*n* = 4), a carer (*n* = 1) and healthcare professionals (*n* = 9). All participants attended one or both workshops in person, except for two participants (one patient and one carer) who preferred to attend individual online co‐design sessions with the research team (A.D. and C.C.).

### 3.1 Workshop Transcript Results

#### 3.1.1 Content Analysis

Personas were used differently in the two scenarios (Tables 1 and 2). In Workshop 2, where participants were considering the needs of patients and providers in ED with a patient such as Bill, who is at risk of escalating distress and agitation, personas were referenced over three times more than in Workshop 1 (57 v 182). While a facilitator was the only speaker to reference the provider persona in Workshop 1, in Workshop 2 providers and facilitators referred to the provider persona equally. The patient persona was referred to more often than the provider persona across both workshops.

**Table 1:** References to personas by persona category.

	Workshop 1		Workshop 2	
Persona	Persona reference count	Persona reference %	Persona reference count	Persona reference %
Patient	50	22%	144	39%
Provider	7	3%	38	10%
No reference	175	75%	184	50%
Total	232		366	

**Table 2:** References to patient and provider personas by speaker group.

	Reference to patient persona				Reference to provider persona			
	Workshop 1		Workshop 2		Workshop 1		Workshop 2	
Speaker	Patient persona reference count	Persona reference %	Patient persona reference count	Persona reference %	Provider persona reference count	Persona reference %	Provider persona reference count	Persona reference %
Facilitator	15	30%	36	25%	7	100%	19	50%
Patient	19	38%	29	20%	0	0%	0	0%
Provider	16	32%	79	55%	0	0%	19	50%
Total	50		144		7		38	

**Figure 1:**
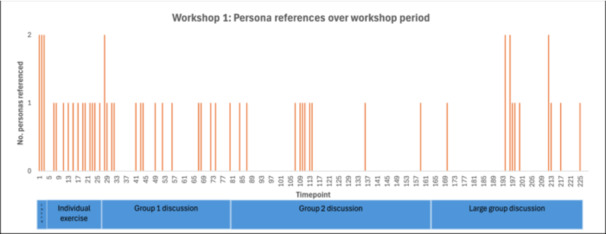
Workshop 1 Persona references over workshop period.

**Figure 2:**
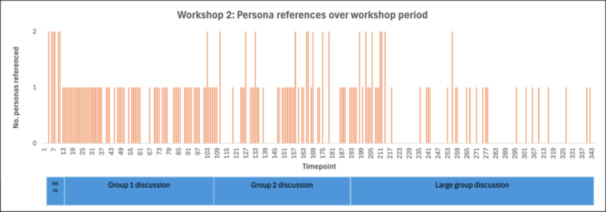
Workshop 2 Persona references over workshop period.

References to the personas during the workshops are presented chronologically and against each workshop program component (Figures 1 and 2). The timelines show that references to the personas were clustered in Workshop 1 at the commencement of the workshop, whereas the personas were used more often during small group discussions and activities in Workshop 2.

#### 3.1.2 Deductive Analysis

**Figure 3:**
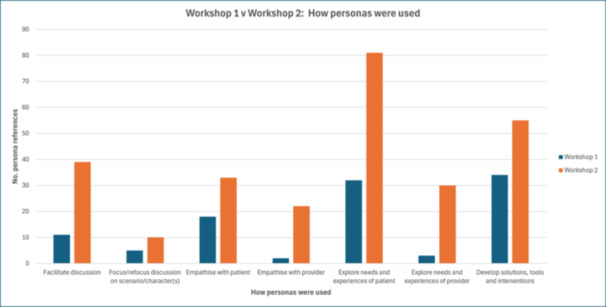
How personas were used in the workshops.

Personas were used in different ways across both workshops: to facilitate and refocus discussion on the scenario and personas; to empathise with both patients and providers; to explore needs and experiences of patients and providers; and to stimulate the development of potential solutions, tools and interventions (Figure 3). Across both workshops, personas were most often used to explore the needs and experiences of patients and to develop solutions, tools and interventions. These persona functions are described below, using participant quotes in illustration.

The facilitators used the persona dyads to facilitate workshop discussions, including introducing activities and exploring participant responses. Facilitators also intentionally leveraged personas as a tool to focus the conversation on patient needs and to guide the group back to the topic.

You were saying before that Ana may be so stressed that she doesn't have words to use to get help. What are some other ways to get help if she doesn't have those words?

Facilitator A, Workshop 1

The personas were used by participants to empathise with patients by describing how they feel about the patient persona, placing themselves in the persona's situation, and articulating the emotions they believe the persona would feel [33]. Participants also validated feelings through statements of understanding or by sharing a similar experience from their own lives.

People just need to understand that mental health is just as bad as physical health. Like if her daughter was having a heart attack, you're not going to leave [Ana] in the emergency room by herself… [Ana's] going through a mental health crisis, why is [Ana's mother] leaving her by herself in that environment?

Patient participant A, Workshop 1

Empathy was also evident in this exchange between participants in Workshop 2:

See, Bill is over‐drinking because he wants to stop everything… That's why people keep on drinking because they can't stop thinking. They drink more, they drink more, they drink more, they drink more, they think. So he has to stop thinking.

Patient participant C, Workshop 2

OK, so Bill needs something to help him stop thinking.

Facilitator A, Workshop 2

Would it also help redirecting that thinking?

Provider participant B, Workshop 2

Distraction.

Patient participant A, Workshop 2

Redirecting or stop thinking. Distraction, what kind of a distraction is there? I've been to ED multiple times, there is no distraction. Actually, it is so quiet and then you just start hearing yourself louder and louder.

Patient participant C, Workshop 2

Drawing on the patient persona, the facilitator reframed a patient participant's insight, that ‘Bill needs something to help him stop thinking’ as a design problem, prompting other participants to offer strategies such as redirection and distraction, while a patient participant grounded these proposals in the lived reality of the ED environment.

Likewise, participants empathised with the provider personas, as shown through expressions of emotion and understanding. In the following exchange, two providers used the personas to address professional roles and responsibilities and to voice divergent perspectives.

OK, I'm going to say something very controversial. I know I'm going to be in trouble with all of you guys from ED but I think it's about mindset also and I think it's about ownership and for Gina to go, ‘This is my patient until Bill leaves ED’. And I think I see that all the time, I've spent enough time now in the Emergency to realise a lot of ED staff go, ‘I don't want this patient, to me it's not my kind of patient’.

Provider participant G, Workshop 2

This mindset that you are talking about exists but it's not in ED, it's about people in general. So anyone who's annoying them, you're going to find annoying. Bill, while it is that he's come in unwell, treating Bill might be annoying because Bill is agitated, Bill is attacking people.

Provider participant F, Workshop 2

Workshop participants engaged with the personas throughout their discussions, using them as prompts to explore the diverse needs, challenges, and lived experiences of both patients and providers. The personas helped structure conversations, enabling participants to consider perspectives they might not otherwise have imagined and reflect on emotional and contextual factors influencing each persona's experience.

[Ana] might be someone who might do well with early intervention supports. She needs crisis counselling, she needs social supports… Sit with her, ask what she needs, link her to services, so that we can assess people's needs quickly.

Provider participant D, Workshop 1

A sense of control, is the big one that, I think, could be provided in other ways as well. [Bill's] got nothing on him, there's nothing he can call his own except his clothes.

Patient participant B, Workshop 2

What I think that [Gina] needs that she doesn't have, and I don't think she's gonna find this, is time. I think she needs time. She needs time to assess Bill, figure out what's going on… All that's got to be happening concurrently without the ability to access the safe space and the resources that she needs.

Provider participant C, Workshop 2

A Workshop 1 exchange shows how the facilitator and participants used the personas to probe the variability and complexity of patient needs:

So can I ask something on process? If Ana was to come back to the ED multiple times, is it likely that her needs will be fairly consistent in the waiting room? Like she would need similar things each time or would she need different things?

Facilitator C, Workshop 1

In personal experience, I think needs are quite different every time I present to the ED. I'm not sure if people might present with similar things but for me there could be different things that are happening. So it would be nice if there was a set Ana care package but…

Patient participant A, Workshop 1

So that's what made me ask that question. Because I was thinking like if you go on a flight, if you've got a frequent flyer number for example, you can pre‐order that you always prefer a vegetarian meal and always prefer an aisle seat for example, so the airline knows what you always want. If it's not consistent, that wouldn't work, but if it was consistent your electronic health record could have preferences on it so when you came in which package is for you.

Facilitator C, Workshop 1

I feel like for me personally, it's not like that because the distress is different. If I'm anxious, for me what helps is raising my heartbeat by like doing a jog on the spot and I trick my brain into thinking my heart is racing due to that jog on the spot and not my anxiety. But that's not helpful when I'm feeling suicidal. It could be the same level of distress, like I could be feeling a 10 of anxiety, I feel like I'm going to have a panic attack, and feeling a 10 in terms of depression and I want to kill myself, running on the spot will not help with that. I feel like the thing with mental health is that a lot of people have lots of different conditions so it's hard to specialise one thing for them because they could be presenting to the ED for different reasons.

Patient participant A, Workshop 1

That's where we fall over because we go acuity and then push complexity aside.

Provider participant B, Workshop 1

Here the facilitator's ‘*frequent flyer*’ analogy tested an assumption that a returning patient's needs would be consistent; the patient participant drew on their own experience to complicate this, and the provider's closing reflection, that services tend to prioritise acuity over complexity, shows how persona‐supported dialogue surfaced a systemic tension.

Personas served as catalysts for creative thinking, stimulating the development of potential solutions, tools and interventions by grounding the ideation process in the needs, challenges, and contexts presented in each persona. By referring back to what each persona might think, feel, or experience, participants generated ideas that were targeted and person‐centred.

I mean, for me, there was a light bulb moment around therapeutic intervention. I don't think as emergency nurses, we're very equipped to deal with that immediate, therapeutic intervention for what was described as distress levels… we would need training and gain some expertise from both consumers and specialists in the area of how a nurse in an emergency department could help with those distress levels.

Provider participant A, Workshop 1

Like, for the patient, [Ana] might be like described as shy, and she's going through distress. It might have already taken all her effort to come to the ED to get help. So although I also understand on the staff's perspective that it's impossible to be checking in on everyone how they're doing like every so now and then, but like maybe, a way to communicate whether that's like, like a card that you hold up or something because sometimes you just you're not verbal in that situation and you can't physically ask for help. So it might be like another way that's possible, like for her to communicate herself would be helpful.

Patient participant A, Workshop 1

Having a more integrated approach.… one of the issues is going to be all these things happen at different times and part of the strategy to battle that would be to have a unit in which the clinicians were housed so that they are in a similar place at similar times. So that even if it is that, say Bill comes in and needs assessment by all these separate teams, the idea would be that the delay between them would be minimised.

Provider participant F, Workshop 2

In the following discussion sequence, the persona kept the discussion grounded in Ana's distress while a patient and a provider built on one another's contributions. The provider's response, noting that they would not themselves have proposed the strategy the patient described, surfaced experiential knowledge that prompted a provider to reconsider their assumptions.

I guess patients sometimes don't really know their own distress levels that well. So they could have support in knowing a little bit about what strategies they could use to calm themselves and maybe we could have a list of common strategies?… Because [Ana] started out like not knowing what strategies to use because she's never been taught them. The nurse could be the one who actively offers possible solutions.

Patient participant A, Workshop 1

I think that's linking to my frustration. We don't have any, we don't think about those things as a therapeutic intervention to address and link it to that distress scale.

Provider participant A, Workshop 1

I feel like in terms of distress it's very different what you do in different situations as well. Like, if you're in very high distress, sometimes just being distracted from your thoughts is helpful. For example, the ice cold water, dumping your hands in ice cold water that helps like recentre you and helps ground you. Whether that's, like giving CBT is not helpful in that moment where your thoughts are already seeing and everything is sensory overload.

Patient participant A, Workshop 1

That's good, so we would pick that up. I would never offer ice water to anyone, ever, so just understanding how you can reset yourself because that really is a difficult issue for us.

Provider participant A, Workshop 1

### 3.2 Survey Results

Across the two co‐design workshops, 15 ‘Persona dyad evaluation’ surveys were completed by 11 of 14 unique workshop participants (patients: 6 surveys from 4 individuals; ED providers: 9 surveys from 7 individuals). Patient respondents included two females, one male and one non‐binary, with an average age of 32 and half of participants spoke a language other than English at home. Provider respondents included three males and four females with an average age of 45, and comprised five nurses and two doctors. The patient and carer participants who attended the individual online co‐design sessions were not asked to complete the PPEET survey which was administered to evaluate workshop dynamics and broader project engagement.

Perceived utility and acceptability of the persona dyads as a person‐centred approach was high, with positive responses from both patients and providers to questions on the helpfulness of personas for imagining and understanding both patient and provider experiences and perspectives (Table 3).

Designing change is always very difficult in the ED as there are so many dependencies… the [persona], however, was useful in ensuring the patient remained in the centre of all discussions when discussing potential ‘change' ideas in the workshop. For example, a ‘purpose‐built’ mental health room may not actually be what's required, rather a multipurpose/multiuse sensory room that was as adaptive and dynamic as the consumer.

Provider participant, Workshop 1

[The persona] does help others see the patient as a person (outside of a patient).

Patient participant, Workshop 2

Great reference point. [The persona] humanises the experience and discussion.

Provider participant, Workshop 2

**Table 3:** Persona dyad evaluation survey^a^ and PPEET responses.

	Workshop 1			Workshop 2		
	Overall	Consumer	Provider	Overall	Consumer	Provider
*Utility of persona dyads in co‐design workshops*						
	Mean (SD)^c^	Mean (SD)	Mean (SD)	Mean (SD)	Mean (SD)	Mean (SD)
Q3 How helpful did you find the characters and scenarios for *understanding problems* in the Emergency Departments?	4.8 (0.4)	4.7 (0.6)	5.0 (0.0)	4.4 (0.5)	4.7 (0.6)	4.3 (0.5)
Q4 How helpful did you find the characters and scenarios for *identifying potential solutions* in the Emergency Departments?	4.7 (0.5)	4.7 (0.6)	4.7 (0.6)	4.2 (0.7)	4.3 (0.6)	4.2 (0.8)
Q5 How helpful did you find the characters and scenarios for *designing changes* in the Emergency Departments?	4.8 (0.4)	5.0 (0.0)	4.7 (0.6)	4.1 (0.6)	4.3 (0.6)	4.0 (0.6)
Q8 Was it helpful to imagine *both sides of the ED experience*, i.e., both the patient receiving care and ED staff member delivering care?	4.8 (0.4)	4.7 (0.6)	5.0 (0.0)	4.4 (0.5)	4.7 (0.6)	4.3 (0.5)
*Sharing views and perspectives* b						
	Mean (SD)^d^	Mean (SD)	Mean (SD)	Mean (SD)	Mean (SD)	Mean (SD)
Q7 I was able to express my views freely.	4.8 (0.4)	5.0 (0.0)	4.7 (0.6)	4.6 (0.5)	4.7 (0.6)	4.5 (0.5)
Q8 I feel my views were heard.	5.0 (0.0)	5.0 (0.0)	5.0 (0.0)	4.4 (0.5)	4.3 (0.6)	4.5 (0.5)
Q9 A wide range of views on the topics discussed was shared.	4.8 (0.4)	5.0 (0.0)	4.7 (0.6)	4.4 (0.5)	4.7 (0.6)	4.3 (0.5)
*Utility of persona dyads in co‐design workshops*						
	Yes/No/Uncertain	Yes/No/Uncertain	Yes/No/Uncertain	Yes/No/Uncertain	Yes/No/Uncertain	Yes/No/Uncertain
Q6 Did the characters help you to *understand the experience of the consumer* in the Emergency Department?	Yes (5); Uncertain (1)	Yes (2); Uncertain (1)	Yes (3)	Yes (8); Missing (1)	Yes (2); Missing (1)	Yes (6)
Q7 Did the characters help you to *understand the experience of the ED staff member* in the Emergency Department?	Yes (4); Uncertain (2)	Yes (2); Uncertain (1)	Yes (2); Uncertain (1)	Yes (8); No (1)	Yes (3)	Yes (5); No (1)
Q12 Do you think the characters and scenarios helped workshop group members *communicate with each other*?	Yes (6)	Yes (3)	Yes (3)	Yes (9)	Yes (3)	Yes (6)
Q13 Do you think the character cards helped you to *consider the perspective* of that fictional person?	Yes (6)	Yes (3)	Yes (3)	Yes (9)	Yes (3)	Yes (6)
Q14 Do you think the character cards *changed the way you perceived care* in the Emergency Department?	Yes (2); No (2); Uncertain (2)	Yes (1); No (1); Uncertain (1)	Yes (1); No (1); Uncertain (1)	Yes (2); No (4); Uncertain (2); Missing (1)	Yes (2); Uncertain (1)	No (4); Uncertain (1); Missing (1)

^a^ In the workshop and evaluation survey, the term ‘persona' was replaced with ‘character.’

^b^ Selected PPEET responses.

^c^ Very helpful (5), Moderately helpful (4), Neither helpful nor unhelpful (3), Moderately unhelpful (2), Very unhelpful (1).

^d^ Strongly agree (5), Agree (4), Neither agree nor disagree (3), Disagree (2), Strongly disagree (1).

Participants also reported that persona dyads were a useful participatory tool for communicating with each other and identifying changes and solutions in ED, with the workshop environment enabling open expression and diverse perspectives (Table 3).

It was more challenging to consider two perspectives simultaneously as opposed to just one however it is more realistic when creating impactful resolutions.

Patient participant, Workshop 1

As a group we were able to not only identify quick solutions (low‐hanging fruit), but map more longer term/systems solutions too.

Provider participant, Workshop 1

Multiple times, the group referred to the character cards/scenarios. We could then extrapolate/have more in‐depth discussion from that point.

Provider participant, Workshop 2

Shifts in how the personas helped participants to perceive care were mixed (Table 3), suggesting personas may prompt reflection but not uniformly change perceptions, particularly among providers in the second workshop.

I feel I still have the same perception of care in the ED but perhaps a better understanding of consumer POV [point of view].

Provider participant, Workshop 2

Workshop participants were also asked questions about the presentation of the persona dyad cards (refer Supporting Information S5: Appendix 5). All participants responded that the personas and scenarios were realistic. Most respondents thought the persona cards had the right level of health detail; views were more mixed about the level of personal detail presented.

I found the ‘character' perspective a valuable resource in visualising each scenario presented to me. Each character had enough detail to imagine them as real people which enabled me to contribute personal ideas to the workshop.

Patient participant, Workshop 1

### 3.3 Facilitator Reflections

The facilitator and three research team members who attended Workshop 1 observed that the persona dyad, especially the patient persona (Ana), was central to guiding discussions, enabling participants to explore sensitive issues without disclosing personal information. Across activities, participants frequently referenced Ana's emotions and needs, often pointing to the persona cards or speaking about ‘someone like Ana’ to frame their ideas. Over the workshop period, patient participants appeared to feel increasingly comfortable sharing their own experiences.

The facilitator and two of the four research team members in attendance observed that the persona dyad (Bill and Gina) was actively and consistently used throughout Workshop 2; participants regularly drew on the personas' backgrounds and interests to justify ideas and shape care considerations. Team members also noted variations in how participants connected with the personas. One provider, for example, gestured to the persona card repeatedly while speaking. One patient participant appeared to strongly identify with the patient persona, and their comments increasingly referenced personal experiences as the workshop progressed; the other patient participants mainly referenced the persona only.

### 3.4 Patient and Provider Co‐author Reflections (Box 2)

Box 2 **Patient and provider co‐author reflections.**

Patient author

The use of persona dyads transformed the workshops from daunting tasks to collaborative spaces where I empathised with provider experiences and my personal experiences felt both valued and protected.

The personas provided a third person starting point, which served as an icebreaker that helped bypass my initial discomfort of sharing deeply private experiences in a room full of strangers and established rapport that eventually allowed for more disclosure of personal experiences. Upon learning about traditional EBCD methods, such as filming the patient participant, I found the persona cards to be an innovative and less confronting way to share lived experiences, mitigating concerns about confidentiality and potential biases (e.g., social desirability bias). One issue that I did encounter with utilising the persona cards was when a character, in my case Bill, diverged significantly from my own journey; it felt intrusive and inappropriate to speculate his needs when I haven't walked in his shoes.

I felt a definitive strength of the workshops was the inclusion of provider personas, rather than solely focussing on patient experiences and views. Inclusion of both patient and provider perspectives doesn't just empower both sides equally, it also reminds us that we are all navigating the same system from different yet equally important angles. Provider personas also provided a space to allow staff to articulate their own needs and systemic constraints (e.g., workload and burnout) without fear of overshadowing patient voices. Seeing the provider's side helped us gain insight into their busy world, their needs, their heavy workloads, and their own stresses and see them as humans beyond their professional roles. The dyadic approach not only achieved the ‘humanise’ and ‘contextualise’ goals of the personas, but also ‘illuminated’ more practical goals and solutions, ensuring the workshop outputs were not just idealistic, but operationally feasible.

Provider author

I found that the persona dyads created a safe and constructive way to reflect on clinical experiences while keeping the focus on the patient. This exercise enabled me to articulate challenges and constraints that are often difficult to name directly in the presence of a patient. I was able to acknowledge the emotional impact and complexity inherent in ED practice. At the same time, referring to the patient personas reminded me to see the patient beyond their ED presentation and to really appreciate how the environment, interaction with clinical staff and system pressures shape their experience. I noticed that using personas allowed me to be more honest in conversations, which I think allowed both myself and patients to contribute in a safer way. To this end, the personas helped me humanise the encounters we were discussing, contextualised them within real‐world ED challenges, and highlighted shared priorities that may have remained unspoken without this tool.

Provider author

Participating in co‐design using persona dyads was initially an unfamiliar approach for me as a provider, but it proved highly valuable. The personas brought humanity back to a complex and vulnerable patient cohort who are all too often spoken about in abstract terms in hospital settings, for example, ‘they’, ‘them’, ‘patients’. Referring to ‘Ana’ or ‘Bill’ rather than ‘the patient’ encouraged me to engage more deeply with their experiences, history and emotions. Their fictional nature created a sense of psychological safety for all participants involved, which allowed us to speak openly and candidly about the realities, and clinical and operational challenges of care provision in an ED setting.

My engagement with the personas deepened over the workshops. In the first, I was orienting to the process and the scenario. ‘Ana’ reflected a common ED presentation that realistically, in practice, may be reprioritised in the waiting room or streamed to a lower‐acuity model of care. While important, this felt like a relatively straightforward presentation that typically does not require significant ED resourcing. In contrast, ‘Bill's’ presentation was more complex and closely aligned with scenarios I frequently encounter in clinical practice, which made it easier to empathise with, and engage more actively in discussion for.

### 3.5 Framework

**Figure 4:**
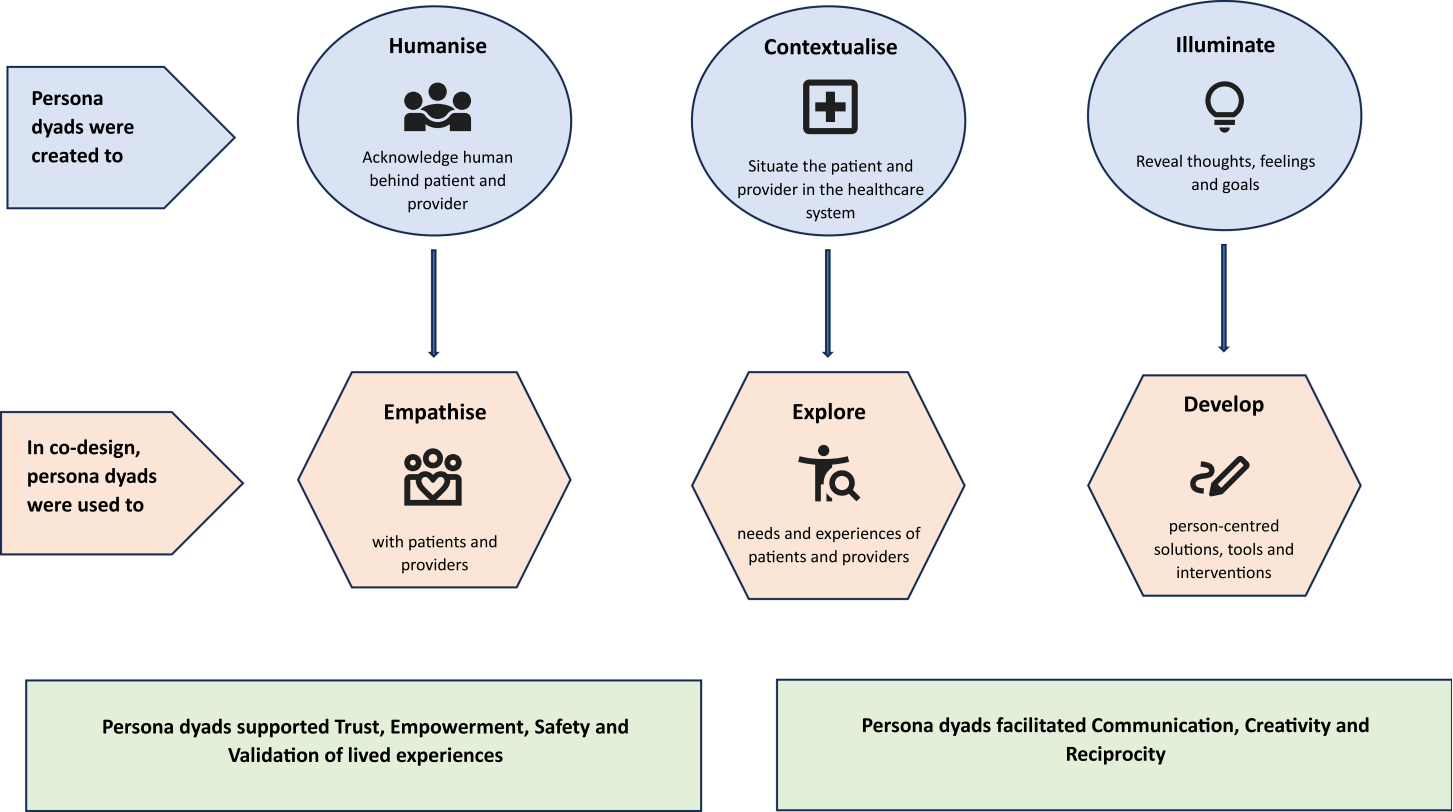
Framework: Using persona dyads to empathise, explore and develop person‐centred solutions in co‐design workshops.

A visual representation of the themes revealed in Part 2 [28] and these results (Part 3) is provided in Figure 4.

## 4 Discussion

This study explored the use of patient/provider persona dyads, created with input from patients and providers, in two co‐design workshops aimed at improving ED care for people presenting with mental health concerns. We designed persona dyads to *humanise*, *contextualise* and *illuminate* the lived experiences of patients and ED providers. During the co‐design workshops, the persona dyads created conditions that supported *trust*, *empowerment*, *safety* and *validation of lived experience*, and facilitated *communication*, *creativity* and *reciprocity*; key enablers of inclusive co‐design, especially in contexts characterised by entrenched power asymmetries such as mental health care [24, 34, 35, 36, 37, 38, 39]. This study contributes to the co‐design and persona literature by demonstrating the value of patient/provider dyads, rather than standalone patient personas, enabling participants to hold both perspectives simultaneously and design solutions with balanced empathy.

Persona use was less frequent in Workshop 1 than Workshop 2. In Workshop 1, we observed that the patient participants shared similarities with the patient persona (Ana) and referred more often to analogous lived experiences, suggesting they became more comfortable as the workshop progressed. Similarly, the provider participants in Workshop 1 were all nurses, as was the provider persona (Darius), and discussed the clinical scenario from a lived clinical perspective. We speculate that the provider participants in Workshop 1 referenced the patient persona to maintain patient participant safety. In Workshop 2, there were more frequent persona references and a stronger emphasis on the provider persona. The clinical scenario of Bill/Gina may have prompted greater reliance on the personas as participants and facilitators navigated more complex patient, sociopolitical and professional dynamics.

### 4.1 Humanising Experiences and Fostering Empathy

A central goal of the persona dyads was to *humanise* both patient and provider experiences. Across both workshops, participants connected and engaged with the emotional vulnerability and personal contexts represented in the personas, frequently referring to their feelings and needs. This humanising effect aligns with the core purpose of traditional EBCD trigger films, which use patient narratives to cultivate empathy and centre lived experiences [21]. In our study, the persona dyads generated empathy without requiring disclosure of personal stories or the use of filmed interviews, which can raise ethical concerns in mental health contexts and limit interview participation [20]. In survey feedback, one patient participant reported that personas help others ‘*see the patient as a person*’. Workshop transcript analysis indicated that the dyads facilitated empathy through not only presenting the patient but also the provider, an often under‐represented perspective in literature reporting co‐design.

This reciprocal empathetic engagement contributed to the development of *trust* within the workshops. We observed that patient participants disclosed more personal information with the persona as the workshop proceeded, and in survey responses all participants agreed that they were able to express views freely. Grounding discussions in fictional characters, rather than personal histories, is particularly valuable when working with priority populations or mixed groups where power asymmetries may inhibit open sharing [38]. Importantly, empathy extended toward both sides of the care relationship. Participants were able to reflect not only on patient experiences but also on provider needs and concerns. In survey responses, all participants agreed that it was helpful to imagine both sides of the ED experience. This balanced empathy is particularly important in co‐design where dual perspectives are often present but not always equally explored.

### 4.2 Contextualising Care and Promoting Shared Understanding

The persona dyads were intentionally designed to *contextualise* patient and provider experiences within the broader ED environment, adopting a systems‐based view. Participants used the personas to explore system constraints, workflow pressures, environmental stressors and complexities influencing care delivery, facets often unrepresented when the patient perspective is the only one visible. Facilitators and participants used the personas to refocus discussion on both the subject and the context. For example, during Workshop 2, when there was speculation about what a patient might be thinking in that scenario, a participant with lived experience stated clearly, ‘*See, Bill is over‐drinking because he wants to stop everything*’.

This contextualising function supported enhanced *communication*. In survey responses, one provider participant described the personas as a ‘*Great reference point*’ and all participants responded ‘Yes’ to the question ‘Do you think the characters and scenarios helped workshop group members communicate with each other?’. The chronological mapping of transcript data showed that persona references were particularly frequent during group discussions, indicating the value of personas as a tool to establish shared understanding, a foundation for constructive collaboration.

### 4.3 Illuminating Needs and Driving Person‐Centred Innovation

Persona dyads were also intended to *illuminate* the thoughts, goals and challenges of patients and providers in critical care moments. Participants proposed ideas that directly responded to persona needs, generating individual‐level solutions that addressed immediate comfort and safety, such as quiet, low sensory spaces, as well as longer‐term system improvements to care pathways, such as integrated care team models. We found that the persona dyads enabled the generation of strengths‐based and person‐centred solutions, which are central to mental health care [11, 39]. For example, a patient participant in Workshop 1 suggested a card system as a way for Ana ‘*to communicate herself*’ if she is finding it difficult to talk, acknowledging staff can't be ‘*checking in on everyone*’. In their survey response, a provider participant in Workshop 1 reflected, ‘*Designing change is always very difficult… a “purpose‐built” mental health room may not actually be what's required, rather a multipurpose/multiuse sensory room that [is] adaptive and dynamic*’. Personas facilitated *creativity* and imaginative thinking by serving as visual storytelling devices [22]. Environments which encourage creativity have been linked to more robust research and development efforts, driving successful innovation outputs and better patient outcomes [40]. Personas also supported *reciprocity*; evident when participants exchanged experiences and ideas with openness and generosity, often drawing connections between their own lives and those of the personas.

The persona dyads contributed to workshop *safety*. Several patients used the personas to speak indirectly about experiences that might otherwise have been distressing or challenging to reveal in a mixed group. For example, one patient participant in Workshop 2 shared, ‘*That's why people keep on drinking because they can't stop thinking. They drink more, they drink more, they drink more, they drink more, they think*’. Providers used personas to challenge professional roles, “*I know I'm going to be in trouble with all of you guys from ED but I think it's about mindset also and I think it's about ownership and for Gina to go, ‘This is my patient until Bill leaves ED*’” (Workshop 2). In this way, the personas appeared to shield participants from perceived personal criticism, acting as a mechanism to explore sensitive topics while preserving emotional and psychological safety [41].

Importantly, the persona dyads supported *empowerment* by articulating strengths, preferences and goals rather than framing patients solely in terms of symptoms or risk. This was evident in workshop discussion comments from patients (e.g., ‘*A sense of control, is the big one that I think*’ Workshop 2) and providers (e.g., ‘*Sit with her, ask what she needs, link her to services*’ Workshop 1). This strengths‐based approach counters deficit‐based narratives common in mental health contexts [42]. Personas were also used by participants to *validate lived experiences*; in survey responses, workshop participants responded that they felt heard and the personas were realistic.

### 4.4 The Facilitator's Role

Facilitation was central to how the persona dyads functioned. Facilitators used the personas to introduce activities, to refocus discussion on patient needs, and to protect patient‐participant safety by directing attention to the fictional character rather than to participants' own histories. They also used the personas to prompt provider reflection, for example by reframing a participant's contribution as a shared design problem, as illustrated in the workshop exchanges above. This is consistent with literature emphasising the facilitator's role in creating psychologically safe and generative conditions for ideation in co‐design [22, 37]. The differing professional and methodological backgrounds of the two facilitators, a nurse with co‐design experience in Workshop 1 and a human factors researcher with co‐design experience in Workshop 2, may also have shaped how the personas were applied in each workshop.

### 4.5 Implications for Co‐Design With Priority Populations

The results of this study suggest that persona dyads may be particularly useful in co‐design initiatives involving priority populations or environments where power imbalances are pronounced. They offer a method that preserves the empathy and person‐centredness of EBCD trigger films while mitigating risks related to identification of individuals, stigma, distress and emotional burden, in perpetuity and susceptible to misaligned reuse [20]. The findings also demonstrate that persona dyads can help level the participatory space. By shifting attention from participants' personal histories to fictional representations, persona dyads support safe and equitable participation of patients and providers. As a facilitation tool, personas encouraged structured yet flexible discussion and stimulated creativity.

### 4.6 The Constructed Nature of Personas

Although the personas were developed from interview data with patients and providers, with the method of construction reported separately [28], the personas remain a constructed account of lived experience: decisions about which attributes, histories and emotions to feature, and which to omit, were ultimately interpretive. A small number of fictional characters cannot represent the heterogeneity of priority populations, and the intersecting identities and experiences of under‐represented groups are therefore only partially represented in Ana, Darius, Bill and Gina.

This raises a question of representational power. When personas are received and discussed by participants who do not share the represented identity, there is a risk more powerful or articulate voices may speak for, reinterpret or override a represented perspective when members of that group are not present to shape how the persona is understood [9, 43, 44]. This has been described as elite capture [45, 46] in some community development literature. Our own data contains a direct instance of this tension. Our patient co‐author reflected that, where the persona (Bill) diverged from their own experience, it ‘*felt intrusive and inappropriate to speculate his needs when I haven't walked in his shoes*’.

Several features of our approach mitigated, but did not eliminate, this risk. The personas were co‐designed with patients and providers, including a co‐author with lived experience of mental illness who also advised on facilitation, and the findings were reviewed with patient and provider co‐authors. We nonetheless acknowledge a residual risk and suggest that future work involve members of the represented communities not only in constructing personas but in shaping how they are received and discussed, for example, through member‐checking of personas with those communities, and facilitation that explicitly names whose voice is, and is not, in the room.

### 4.7 Strengths and Limitations

This study extends existing work on personas in healthcare co‐design [28, 30] by demonstrating that persona dyads can be valuable as a mechanism for supporting reciprocal empathy and collaborative solution development. A strength of this study is the innovative application of persona dyads in adapted EBCD workshops in a mental health context. This approach extends existing work on personas by demonstrating how fictionalised, yet experience‐informed, characters can support psychological safety, balance power, and stimulate creativity in co‐design. The study also draws on multiple data sources, including workshop transcripts, participant surveys, facilitator observations and participant reflections. This triangulation enhances trustworthiness and provides a rich account of how persona dyads functioned as person‐centred and participatory tools in co‐design. The study benefited from the involvement of patient and provider co‐authors, whose insights shaped workshop design, interpretation of findings and contributed to reflexivity. Patient and provider reflections were not sought to verify results, but to reflect on the analysis and offer additional insights [47]. Using the SRQR and GRIPP2‐SF frameworks supported transparency and strengthened methodological rigour.

However, several limitations must be acknowledged. First, the workshops involved a relatively small number of participants from a single Australian health district, which may limit transferability. While purposive sampling ensured diversity across roles, low participant numbers mean findings represent depth rather than breadth. Second, although personas were developed from interview data in a prior study phase, decisions about which elements to include were ultimately interpretive. The persona dyads therefore reflect a constructed version of lived experience rather than an exhaustive portrait [28]. Because a small number of fictional characters cannot capture the heterogeneity of priority populations, the personas should be understood as illustrative rather than representative, and there is a risk that more powerful voices shape their interpretation when members of the represented groups are absent. Nevertheless, they may offer exemplary knowledge of use to others. Third, facilitator presence, and the structured nature of workshop activities, may have influenced how personas were used and how openly participants shared views. Fourth, participants who chose to attend the workshops may have been more positively disposed toward collaborative methods, meaning findings may not reflect the experiences of those less comfortable with co‐design or narrative‐based tools. Finally, the analysis of persona dyads does not provide evidence of effectiveness but it does provide an early indication of their value in co‐design [2].

## 5 Conclusion

This study demonstrates that persona dyads can enable empathetic and inclusive involvement of both patients and providers in the co‐design of mental health ED care. As health systems and researchers increasingly turn to co‐design to address health disparities, enhance care experiences and drive service quality improvements, persona dyads may offer a valuable addition to the methodological toolkit, particularly in contexts characterised by stigma, complexity or structural inequities. Personas may also function effectively as an accelerated EBCD or co‐design approach, helping establish understanding, empathy and person‐centred design principles when engagement with participants is necessarily targeted or short‐term. Future research could examine how persona dyads perform in other clinical settings and priority populations and evaluate impacts of persona‐informed changes on patient and provider experiences and outcomes.

## Author Contributions

**A. Dominello:** conceptualization, methodology, formal analysis, writing – original draft, writing – review and editing. **S. Lu:** validation, writing – review and editing, writing – original draft. **A. De Los Santos:** validation, writing – original draft, writing – review and editing. **M. Murphy:** validation, writing – original draft, writing – review and editing. **R. Clay‐Williams:** conceptualization, methodology, funding acquisition, writing – review and editing, supervision. **C. Cheek:** conceptualization, supervision, methodology, writing – review and editing.

## Ethics Statement

This study was performed in line with the principles of the Declaration of Helsinki. The MyED project obtained ethics approval from the Local Health District Research Ethics Committee (REF: 2022/PID02749‐2022/ETH02447). All names were anonymised with codes to ensure participant privacy. Patient participants and provider participants who attended the workshops in their personal time, were compensated for their contribution with an e‐voucher.

## Consent

All participants provided written or electronic consent to participate in the study.

## Conflicts of Interest

The authors declare no potential conflicts of interest with respect to the research, authorship, and/or publication of this article. A.D. is a PhD student and this study is a component of their thesis.

## Supporting information

Supporting File 1

Supporting File 2

Supporting File 3

Supporting File 4

Supporting File 5

## Data Availability

All data generated or analysed during this study are included in this published article and its Supporting information files.
