# Supplementary material for: “There Was a Light Bulb Moment”: Using Persona Dyads to Empathise, Explore and Develop Person‐Centred Solutions in Mental Health Service Co‐Design
Source: Health Expect. 2026 Aug 4;29(4):e70797. doi: 10.1111/hex.70797 (PMC13436128; doi:10.1111/hex.70797)
Supplement: Supplementary file 1 [file HEX-29-e70797-s001.docx]

**Appendix_4 - GRIPP2 Short form**

| **Section and topic** | **Item** | **Reported on page No** |
| --- | --- | --- |
| 1: Aim | Report the aim of PPI in the study | Introduction, pp 3-4 |
| 2: Methods | Provide a clear description of the methods used for PPI in the study | Methods, pp 7-8 |
| 3: Study results | Outcomes - Report the results of PPI in the study, including both positive and negative outcomes | Results, pp 12 - 15 |
| 4: Discussion and conclusions | Outcomes - Comment on the extent to which PPI influenced the study overall. Describe positive and negative effects | Discussion, pp 19 - 20 |
| 5: Reflections/critical perspective | Comment critically on the study, reflecting on the things that went well and those that did not, so others can learn from this experience | Discussion, pp 19 - 20 |
