# Supplementary material for: “There Was a Light Bulb Moment”: Using Persona Dyads to Empathise, Explore and Develop Person‐Centred Solutions in Mental Health Service Co‐Design
Source: Health Expect. 2026 Aug 4;29(4):e70797. doi: 10.1111/hex.70797 (PMC13436128; doi:10.1111/hex.70797)
Supplement: Supplementary file 2 [file HEX-29-e70797-s002.docx]

**Appendix_2 – Analysis codes for persona use in co-design workshops**

| **Code** | **Description / Example quote*** |
| --- | --- |
| Facilitate discussion | Facilitator prompts discussion, seeks response to a question.  For example, *Great. Anybody else got anything else to add to seeing Ana as a person?* |
| Focus/refocus discussion on scenario/character(s) | Facilitator keeps discussion user-focussed and on topic.  For example, *I want you to imagine that you are Bill and what your needs might be if you were Bill.* |
| Empathise with patient | For the patient persona, a participant: expresses feeling about them; puts themselves in their situation and feels their emotions; validates their feelings and communicates their understanding; or shares their experience in parallel with the patient persona.  For example, *In terms of, like, like, how she [Ana] feels like lonely in the waiting room, I think, it's if it's possible for staff to, like, even talk to the caregiver that brought her there, like, her mum so that the mum knows how extreme the situation is. Like you wouldn't leave like you're dying daughter by herself at the hospital if she was like, if it was like a physical condition. So it's, but sometimes when the child says that, the parents don't take it as seriously but if staff could possibly intervene and tell them that they really need someone with them in this moment to support them and advocate for them, it might be helpful and also might help take some load off the staff as well.* |
| Empathise with provider | For the provider persona, a participant: expresses feeling about them; puts themselves in their situation and feels their emotions; validates their feelings and communicates their understanding; or shares their experience in parallel with the provider persona.  For example, *And that that comes back to, as you said, appropriate staffing, having not having one person stretched for an entire week to be 24 hours on call the entire week because they're not going to give better care to, to Bill who's needing it. And Bill’s probably not going to be appreciated being treated by this person who's so tired today and can’t remember Bill’s last name.* |
| Explore needs and experiences of patient | For example, *And initially with him [Bill] we should really recognise he's intoxicated, he's, by the sounds of it, potentially on other drugs as well. So we've gotta meet at a level that we want from him, so calm, introducing calmly, you know, acknowledging and validating who he is, where he is, and we need to work together to get where he wants to go.* |
| Explore needs and experiences of provider | For example, *In terms of time, what we were saying is it's kind of a bit of a challenge and I guess a need for Gina, is that everything needs to happen concurrently from an ED point of view.* |
| Develop solutions, tools and interventions | For example, *There’s something else I wanted to add, it’s what to bring. Because especially if it’s [Ana’s] first time she doesn’t know what she needs to bring. So even if that’s an extra jacket, or her headphones, or a fidget toy or whatever, we could provide a handout of what possible things to put in her bag.* |

* Patient personas: Ana and Bill; Provider personas: Darius and Gina
