# Supplementary material for: “There Was a Light Bulb Moment”: Using Persona Dyads to Empathise, Explore and Develop Person‐Centred Solutions in Mental Health Service Co‐Design
Source: Health Expect. 2026 Aug 4;29(4):e70797. doi: 10.1111/hex.70797 (PMC13436128; doi:10.1111/hex.70797)
Supplement: Supplementary file 3 [file HEX-29-e70797-s003.docx]

**Character workshop questionnaire**

**Thank you for completing this brief questionnaire. We would like to know your opinion about the characters and scenarios that we used in the workshop.**

This questionnaire is voluntary. Please be aware that all of the information you provide will be confidential. This research project has been approved by the Western Sydney Local Health District Human Research Ethics Committee REF: REF 2022/PID02749-2022/ETH02447. Should you have any questions about this questionnaire, please contact Dr Colleen Cheek, email: MyED@mq.edu.au.

1. In what role were you participating in the co-design workshop?

☐ Person with lived experience of receiving care in an Emergency Department for mental illness

☐ Support person of a person who has received care in an Emergency Department for mental illness

☐ Nurse

☐ ED Doctor

☐ Doctor from a different specialty

☐ Other

If Other, please describe _________________________________________________

2. Did you meet with a member of the MyED research team before this workshop to help create the characters?

☐ Yes

☐ No

☐ Can’t recall

3. How helpful did you find the characters and scenarios for **understanding problems** in the Emergency Departments?

☐ Very helpful

☐ Moderately helpful

☐ Neither helpful nor unhelpful

☐ Moderately unhelpful

☐ Very unhelpful

Please explain the reason for your response.

4. How helpful did you find the characters and scenarios for **identifying potential solutions** in Emergency Departments?

☐ Very helpful

☐ Moderately helpful

☐ Neither helpful nor unhelpful

☐ Moderately unhelpful

☐ Very unhelpful

Please explain the reason for your response.

5. How helpful did you find the characters and scenarios for **designing changes** in Emergency Departments?

☐ Very helpful

☐ Moderately helpful

☐ Neither helpful nor unhelpful

☐ Moderately unhelpful

☐ Very unhelpful

Please explain the reason for your response.

6. Did the characters help you to understand the experience of the **consumer** in the Emergency Department?

☐ Yes

☐ No

☐ Uncertain

Please explain the reason for your response.

7. Did the characters help you to understand the experience of the **ED staff member** in the Emergency Department?

☐ Yes

☐ No

☐ Uncertain

Please explain the reason for your response.

8. Was it helpful to imagine both sides of the ED experience, i.e. both the patient receiving care and ED staff member delivering care?

☐ Very helpful

☐ Moderately helpful

☐ Neither helpful nor unhelpful

☐ Moderately unhelpful

☐ Very unhelpful

Please explain the reason for your response.

9. Were the characters and scenarios realistic?

☐ Yes

☐ No

☐ Uncertain

Please explain the reason for your response.

10. Do you think the character cards had the right level of personal detail (e.g. age, employment, personality)?

☐ Too much

☐ Just right

☐ Too little

☐ Uncertain

Please explain the reason for your response.

11. Do you think the character cards had the right level of health detail (e.g. medical history)?

☐ Too much

☐ Just right

☐ Too little

☐ Uncertain

Please explain the reason for your response.

12. Do you think the characters and scenarios helped workshop group members communicate with each other?

☐ Yes

☐ No

☐ Uncertain

Please explain the reason for your response.

13. Do you think the character cards helped you to consider the perspective of that fictional person?

☐ Yes

☐ No

☐ Uncertain

Please explain the reason for your response.

14. Do you think the character cards changed the way you perceived care in the Emergency Department?

☐ Yes

☐ No

☐ Uncertain

Please explain the reason for your response.

15. Do you have any other comments you would like to offer about the characters and scenarios we used in the workshop?
16. Are you interested in attending a short interview about the characters and scenario we created in the workshop?

☐ Yes

☐ No

☐ Uncertain at the moment
