# Supplementary material for: “There Was a Light Bulb Moment”: Using Persona Dyads to Empathise, Explore and Develop Person‐Centred Solutions in Mental Health Service Co‐Design
Source: Health Expect. 2026 Aug 4;29(4):e70797. doi: 10.1111/hex.70797 (PMC13436128; doi:10.1111/hex.70797)
Supplement: Supplementary file 4 [file HEX-29-e70797-s004.docx]

**Appendix_File 5 – Presentation of personas survey responses**

|  | **Workshop 1** | | | **Workshop 2** | | |
| --- | --- | --- | --- | --- | --- | --- |
|  | **Overall** | **Consumer** | **Provider** | **Overall** | **Consumer** | **Provider** |
| **Presentation of persona dyad cards** |  |  |  |  |  |  |
|  | **Yes /No / Uncertain** | **Yes /No / Uncertain** | **Yes /No / Uncertain** | **Yes /No / Uncertain** | **Yes /No / Uncertain** | **Yes /No / Uncertain** |
| Q9 Were the characters and scenarios **realistic**? | Yes (6) | Yes (3) | Yes (3) | Yes (9) | Yes (3) | Yes (6) |
|  | **Too much / Just right / Too little / Uncertain** | **Too much / Just right / Too little / Uncertain** | **Too much / Just right / Too little / Uncertain** | **Too much / Just right / Too little / Uncertain** | **Too much / Just right / Too little / Uncertain** | **Too much / Just right / Too little / Uncertain** |
| Q10 Do you think the character cards had the right level of **personal detail** (e.g. age, employment, personality)? | Just right (5); Uncertain (1) | Just right (2);  Uncertain (1) | Just right (3) | Too much (2);  Just right (4);  Uncertain (2);  Missing (1) | Uncertain (2);  Missing (1) | Too much (2);  Just right (4) |
| Q11 Do you think the character cards had the right level of **health detail** (e.g. medical history)? | Just right (3);  Too little (1);  Uncertain (1);  Missing (1) | Too little (1);  Uncertain (1);  Missing (1) | Just right (3) | Just right (8), Uncertain (1) | Just right (2), Uncertain (1) | Just right (6) |
